# Atypical Interplay of Dermatomyositis, Metastatic Caecal Carcinoma, and Connective Tissue Disease

**DOI:** 10.7759/cureus.100665

**Published:** 2026-01-03

**Authors:** Yi Ru Tan, Saileesh Gunasekaran, Melvin Chua

**Affiliations:** 1 Department of General Medicine, Sengkang General Hospital, Singapore, SGP

**Keywords:** colorectal cancer, connective tissue disease, dermatomyositis, metastatic caecal carcinoma, paraneoplastic presentation

## Abstract

This report presents the case of a 71-year-old Chinese female with atypical symptoms leading to the diagnosis of metastatic caecal adenocarcinoma with overlapping connective tissue disease, initially identified as possible paraneoplastic dermatomyositis. The patient, with no significant medical history, was referred for elevated serum creatine kinase (CK) levels and exhibited progressive lethargy, upper limb weakness, and notable weight loss over three months. Clinical examination revealed violaceous rashes and significant muscle weakness, prompting a provisional diagnosis of dermatomyositis. However, extensive investigations, including computed tomography (CT) imaging and colonoscopy, uncovered metastatic disease characterized by peritoneal collections and a caecal tumor. The autoimmune panel indicated elevated anti-ribonucleoprotein (anti-RNP), anti-Sjögren's Syndrome A/Ro (anti-SSA/Ro), anti-topoisomerase I (anti-Scl-70), and anti-Ku antibodies, while key cancer-associated myositis markers were absent. This scenario highlights the complex interplay between malignancy and autoimmunity, emphasizing the necessity for a multidisciplinary approach in diagnostic evaluation. The findings advocate for heightened awareness of malignancy in older adults presenting with inflammatory myopathy, as timely diagnosis and intervention are critical in reducing morbidity and mortality associated with malignancies.

## Introduction

Dermatomyositis (DM) is an idiopathic inflammatory myopathy characterized by symmetrical proximal muscle weakness, elevated serum creatine kinase (CK), and distinctive cutaneous manifestations such as heliotrope rash, shawl-like rash on the back/chest, and Gottron’s papules. It represents one of the strongest malignancy-associated autoimmune syndromes, with cancer risk estimated to be four times higher than in the general population. Most malignancies are identified within one to three years of dermatomyositis diagnosis, supporting its role as a potential paraneoplastic phenomenon [1].

The link between dermatomyositis and malignancy is particularly relevant in older adults, and specific myositis-specific antibodies (MSAs) such as anti-transcription intermediary factor 1-gamma (anti-TIF1-γ) and anti-nuclear matrix protein 2 (anti-NXP2) are known markers of cancer-associated dermatomyositis. In contrast, antibodies such as anti-Mi-2 and anti-melanoma differentiation-associated gene 5 (anti-MDA5) are associated with non-neoplastic subtypes, the latter often linked with interstitial lung disease (ILD).

We report a case of dermatomyositis-like presentation in an elderly woman ultimately diagnosed with metastatic caecal adenocarcinoma, in whom the absence of cancer-associated antibodies and presence of overlap-connective tissue disease antibodies posed significant diagnostic challenges.

## Case presentation

A 71-year-old Chinese female with no prior medical history was referred to the Emergency Department (ED) for evaluation of elevated CK. She had a two-month history of progressive lethargy, proximal bilateral upper and lower limbs weakness, and significant unintentional weight loss of six kilograms over three months. One week of self-administered Traditional Chinese Medicine provided no relief.

On arrival at the ED, she appeared lethargic with mild facial puffiness; systemic examinations were otherwise unremarkable. Laboratory results (Table 1) revealed markedly elevated CK, raised C-reactive protein (CRP) and aldolase, transaminitis, normocytic anemia, and raised erythrocyte sedimentation rate (ESR).

**Table 1:** Laboratory results of serum blood samples

Laboratory Results	Result (unit)	Reference value (unit)
Creatine Kinase (CK)	2623 (U/L)	25-200 (U/L)
Aspartate Aminotransferase (AST)	233 (U/L)	= 35 (U/L)
Alanine Aminotransferase (ALT)	117 (U/L)	= 35 (U/L)
Haemoglobin (Hb)	9.8 (g/dL)	12-16 (g/dL)
Mean Corpuscular Volume (MCV)	83.5 (fL)	78-98 (fL)
Mean Corpuscular Haemoglobin (MCH)	27.0 (pg)	27-32 (pg)
Erythrocyte Sedimentation Rate (ESR)	45 (mm/hr)	<30 (mm/hr)
C-Reactive Protein (CRP)	=4.9 (mg/L)	19.2 (mg/L)
Aldolase	29.8 (U/L)	1.3-6.3 (U/L)

**Figure 1:**
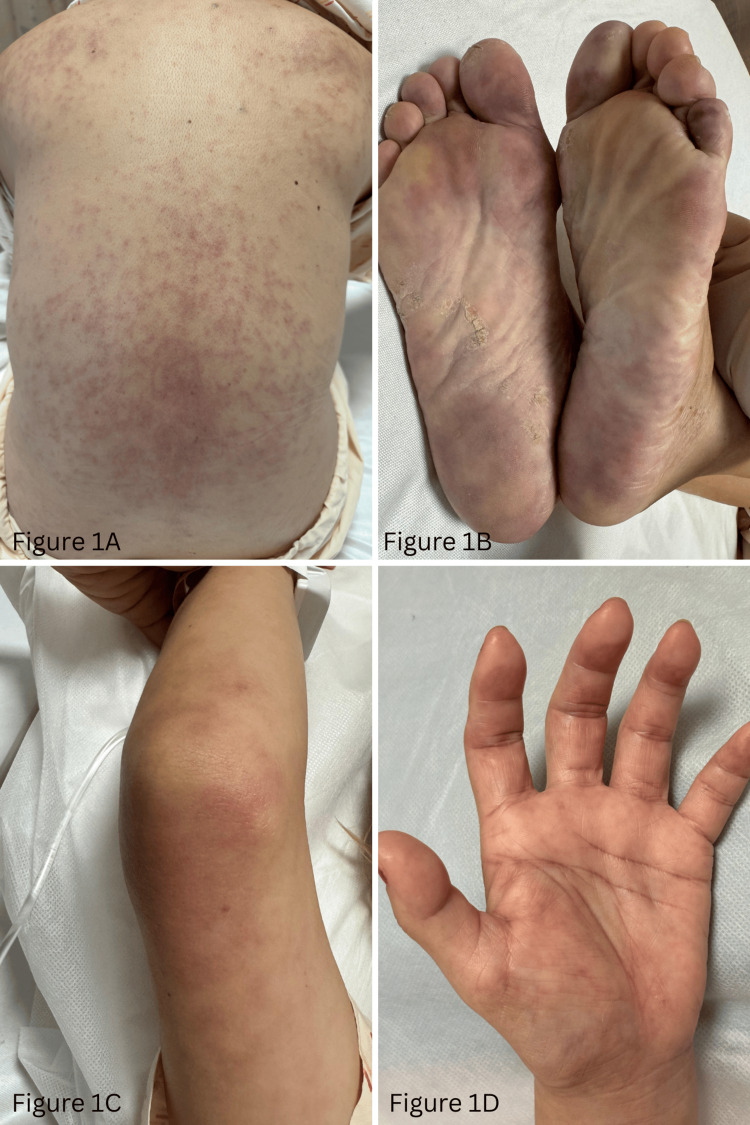
Violaceous rashes over the back, soles of feet, elbow, and hand (1A-1D)

During admission, she was noted to have diffuse violaceous rashes over the back, soles of feet, and elbows and hands (Figure 1A-1D), with acral and truncal distribution atypical for classic heliotrope and Gottron-type dermatomyositis eruptions.

Neurological examination revealed prominent proximal muscle weakness in bilateral upper and lower limbs. A provisional diagnosis of dermatomyositis was made, followed by malignancy screening due to associated systemic symptoms.

Contrast-enhanced computed tomography (CT) of the chest, abdomen, and pelvis demonstrated multiple peritoneal collections, omental thickening, caecal wall thickening, and a hypodense hepatic lesion - all indicative of metastatic disease. Colonoscopy revealed a circumferential obstructing caecal tumour; biopsy confirmed adenocarcinoma. Fine needle aspiration (FNA) of a peritoneal nodule and a right iliac fossa collection both confirmed metastatic adenocarcinoma.

An autoimmune panel (Table 2) revealed elevated anti-ribonucleoprotein (anti-RNP), anti-Sjögren's Syndrome A/Ro antibodies (anti-SSA/Ro), anti-topoisomerase I (anti-Scl-70), and anti-Ku antibodies. Notably, anti-transcription intermediary factor 1-gamma antibody (anti-TIF1-γ antibody) and anti-nuclear matrix protein 2 antibody (anti-NXP2) - markers typically associated with cancer-related myositis - were negative. Anti-neutrophil cytoplasmic antibody (ANCA) testing was negative for cytoplasmic anti-neutrophil cytoplasmic antibody (c-ANCA), anti-myeloperoxidase (anti-MPO), and anti-proteinase 3 (anti-PR3).

**Table 2:** Autoimmune panel

Autoimmune panel	Result (unit)	Reference value (unit)
Anti-myeloperoxidase (anti-MPO)	<2.0 (RU/ml)	<20 (RU/ml)
Anti-proteinase 3 (anti-PR3)	2.2 (U/ml)	<10 (U/ml)
Anti-nuclear antibody (ANA)	>640	<160
Anti double stranded deoxyribonucleic acid antibody (Anti-DsDNA)	11.0 (IU/ml)	<10 (IU/ml)
Cytoplasmic anti-neutrophil cytoplasmic antibody (c-ANCA)	Negative	
Anti-Smith	0.1	<1.0
Anti-ribonucleoprotein (anti-RNP)	1.3	<1.0
Anti-Sjögren's Syndrome A/Ro antibody (anti-SSA/Ro)	2.3	<1.0
Anti-Sjögren's Syndrome B/La (anti-SSB/La)	0.1	<1.0
Anti-topoisomerase I (anti-Scl-70)	2.7	<1.0
Jo-1 antibody	0.0	<1.0
Anti-Ku antibody	40	0-14
Anti-transcription intermediary factor 1-gamma antibody (anti-TIF1-γ antibody)	0	0-14
Anti-nuclear matrix protein 2 antibody (anti-NXP2)	3	0-14
Anti-Mi 2	2	0-14
Anti-Melanoma Differentiation-Associated gene 5 (anti-MDA5)	1	0-14
Anti- HMG-CoA reductase antibody (anti-HMGCR)	11	0-14

**Figure 2:**
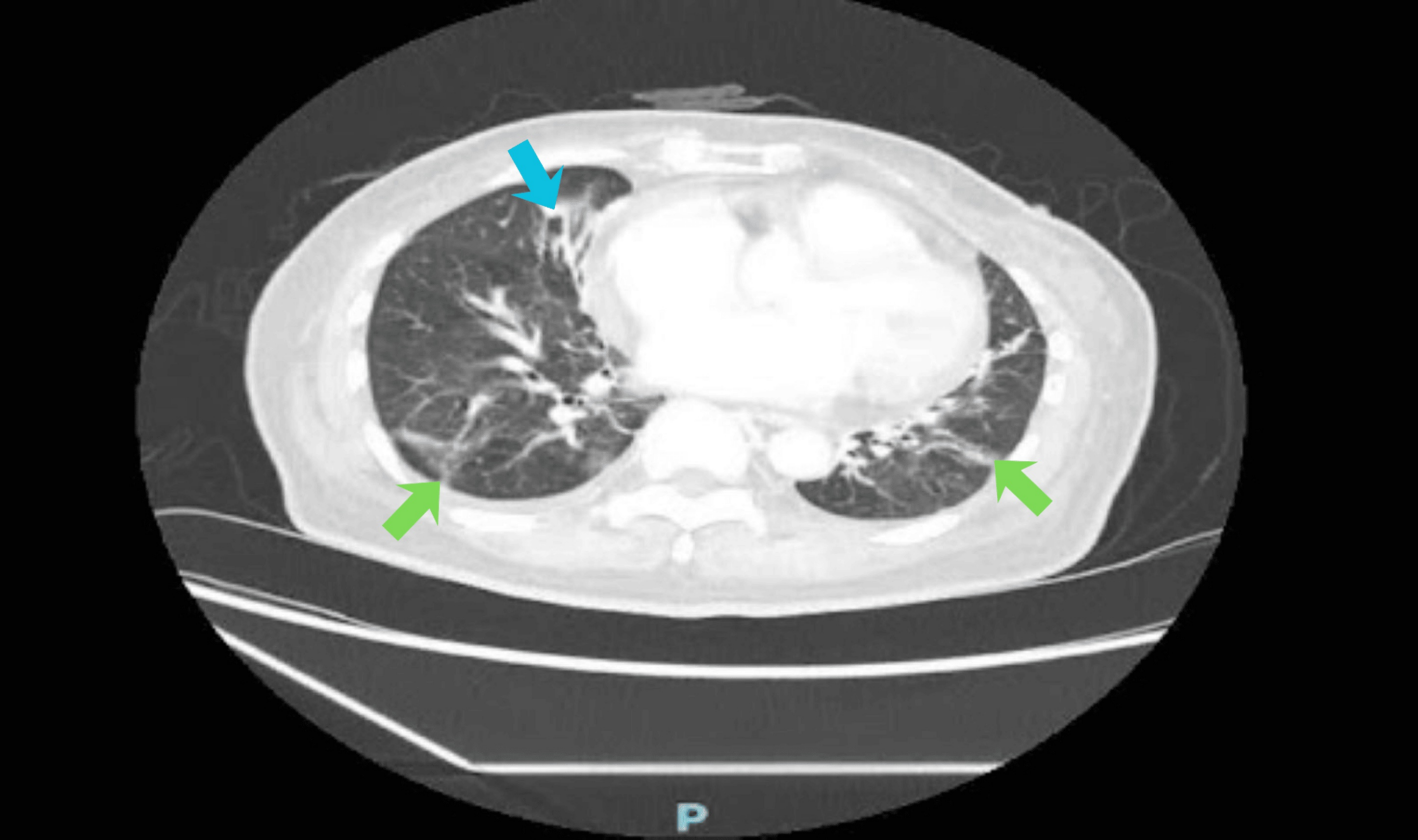
Standard computed tomography (CT) thorax Cross-sectional imaging of lungs; evidence of bronchiectatic lung changes (blue arrow) in the right middle lobe with bilateral patchy ground glass changes (green arrows).

Electromyography (EMG) showed membrane instability and was consistent with inflammatory myopathy. Skin biopsy of the violaceous rash demonstrated histopathologic features consistent with dermatomyositis, with perivascular inflammation and dermal mucin. Direct immunofluorescence (DIF) revealed granular complement factor 3 (C3) and immunoglobulin M (IgM) deposition in blood vessels, with negative staining for IgA, IgG, and fibrin, which is characteristics for dermatomyositis and helps distinguish this entity from vasculitis or lupus erythematosus. CT thorax (Figure 2) noted right middle lobe bronchiectasis and bilateral ground-glass changes, raising suspicion for underlying interstitial lung disease (ILD).

While the patient's presentation initially raised concern for paraneoplastic dermatomyositis, the antibody profile was suggestive of a connective tissue disease overlap. The patient was treated with intravenous immunoglobulin (IVIG) for three days, with notable improvement in proximal weakness, fading, and resolution of rashes over the back, soles of feet, and elbows and hands over one month. Creatine kinase values also showed a decrease in trend to 619 U/L. The patient was initiated on systemic chemotherapy, and under joint rheumatology and oncology follow-up at six to eight weekly intervals for disease surveillance and oncologic treatment.

## Discussion

This case illustrates an atypical presentation of metastatic caecal adenocarcinoma in a previously well older adult, with the initial presentation mimicking paraneoplastic dermatomyositis, given the presence of skin and muscle involvement along with typical systemic features.

The extensive, non-photo-distributed involvement of the trunk and acral sites was atypical for dermatomyositis and initially raised diagnostic uncertainty. 

The skin biopsy DIF revealed results characteristic of dermatomyositis and helps distinguish this entity from vasculitis or lupus erythematosus. Electromyography (EMG) demonstrated findings consistent with inflammatory myopathy, supporting the diagnosis of dermatomyositis. 

Dermatomyositis and colorectal cancer

Dermatomyositis usually presents with proximal muscle weakness and a raised creatine kinase indicative of skeletal muscle inflammation. Adult patients with dermatomyositis face a fourfold increased risk of malignancy compared to the general population, with cancer most commonly diagnosed within three years before or after dermatomyositis onset [1]. Common associated malignancies include ovarian, lung, breast, and colorectal cancers, as well as lymphoma [2-4]. 

Colorectal cancer most often presents with changes in bowel habit, gastrointestinal tract bleeding, weight loss, and other constitutional symptoms. Paraneoplastic syndromes are symptoms caused by the body's immune response to a tumor, which is not a common first presentation of symptoms for colorectal cancer.

Adenocarcinoma is identified as the most common histological type of tumour associated with dermatomyositis-related paraneoplastic syndromes [5,6]. Histology report of biopsy taken from the caecal tumour of our patient revealed adenocarcinoma. Older age is a key risk factor for malignancies in myositis patients, reported in multiple studies, including systematic reviews and meta-analyses [6-9]. Interestingly, our patient had extensive skin involvement and muscle weakness, which were also noted to be important features predictive of malignancy in myositis patients [9].

A study by Opinc and Makowska in 2022 also showed the rapidity of onset of myopathy should encourage greater vigilance in clinicians assessing malignancy risk [10].

Myositis-specific antibodies (MSAs) define clinically distinct dermatomyositis subgroups and aid in risk stratification [11]. Anti-TIF1-γ and anti-NXP2 antibodies are most strongly associated with cancer-associated dermatomyositis, conferring 9.37-fold and 3.68-fold increased cancer risks, respectively [12]. However, these antibodies are present in only approximately 55% of cancer cases occurring within three years of dermatomyositis diagnosis [1]. Myositis-associated antibodies (MAAs), including anti-Ku, anti-Scl-70, anti-SSA/Ro, and anti-RNP, are not specific to myositis and suggest overlap with other connective tissue diseases such as systemic sclerosis or mixed connective tissue disease [11].

Review of the literature indicates that while anti-Ku antibodies are associated with myositis and ILD, they are rarely linked to malignancy [13]. Anti-RNP, anti-SSA/Ro antibodies, and anti-Scl 70 antibodies are antibodies commonly present in autoimmune conditions but lack strong paraneoplastic associations [14]. In our patient, radiographic evidence of ILD alongside anti-Ku positivity further supported the possibility of an undiagnosed connective tissue disease rather than a pure paraneoplastic process. 

Diagnostic challenges with paraneoplastic considerations

The association between cancer and dermatomyositis as a paraneoplastic syndrome has been extensively reported in adults, although the pathogenesis remains unclear at this point.

The interplay between autoimmunity and malignancy in such cases underscores the need for a multidisciplinary diagnostic approach. Mechanistically, cancer-associated dermatomyositis is thought to result from immune responses triggered by tumor neoantigens, somatic mutations in autoantigen genes, and molecular mimicry, supporting a direct link between autoimmunity and malignancy even in the absence of classic cancer-associated antibodies [15,16]. The American College of Chest Physicians recognizes the autoimmune basis of dermatomyositis-malignancy association and recommends comprehensive cancer screening in all adults with new-onset dermatomyositis, regardless of antibody status [17].

Our patient’s antibody panel had significant findings, which provided some diagnostic clarity - anti-TIF1-γ antibody and anti-NXP2 antibody, both documented markers for cancer-associated myositis [18,19], were absent in this case. Our patient’s positive anti-Ku, ant-RNP, anti-SSA/Ro, and anti-Scl-70 antibodies point towards an overlapping connective tissue disorder, providing more weight to a non-paraneoplastic aetiology - specifically, a possible concomitant undiagnosed underlying connective tissue disorder, which was the Rheumatologists’ primary impression. Radiographic evidence of ILD further supported an overlap connective tissue disease phenotype.

The absence of cancer-associated MSAs does not exclude malignancy, especially in older adults with rapid-onset dermatomyositis and constitutional symptoms [12]. Treatment should be multidisciplinary, addressing both the underlying malignancy and the autoimmune process, as tumor-directed therapy can lead to resolution of dermatomyositis symptoms [20]. This case is clinically significant because it demonstrates cancer-associated dermatomyositis with metastatic caecal adenocarcinoma in the absence of classic cancer-associated antibodies (anti-TIF1-γ and anti-NXP2), instead presenting with an unusual overlap antibody profile. This highlights that comprehensive malignancy screening should be pursued in all older adults with dermatomyositis regardless of antibody status, particularly when constitutional symptoms such as weight loss and anemia are present.

## Conclusions

This case underscores the critical need to be aware of the association of an underlying malignancy with dermatomyositis. Diagnostic and therapeutic challenges are made easier by carefully studying the constellation of features in history taking, physical examination, autoimmune panel, and the temporal association between the onset of myositis and the diagnosis of malignancy.

Malignancy in the setting of dermatomyositis is associated with substantially reduced survival compared with dermatomyositis alone, with cancer stage and control being the principal determinants of prognosis. We suggest timely malignancy screening in older patients with new-onset dermatomyositis, especially in the context of rapidly developing symptoms of myositis. Timely recognition and effective oncologic treatment are crucial for improving outcomes.
